# Investigating mediated public engagement with science on the “science” subreddit: From the participants’ perspective

**DOI:** 10.1371/journal.pone.0249181

**Published:** 2021-04-28

**Authors:** Hui Chen, Noriko Hara, Clinton McKay

**Affiliations:** 1 Information & Library Science, Luddy School of Informatics, Computing & Engineering, Indiana University, Bloomington, Indiana, United States of America; 2 Department of Sociology, Indiana University, Bloomington, Indiana, United States of America; Universitá degli Studi di Milano, ITALY

## Abstract

While public engagement with science activities traditionally inhabits physical environments (i.e., museum exhibits), as the Internet becomes more ubiquitous, new types of public engagement with science mediated through information technologies have emerged. Instead of having scientific findings filtered through traditional mediators, scientists have begun to take advantage of social media in order to communicate directly with the general public. This paper focuses on technology mediated public engagement with science in an online environment, specifically the sub-Reddit called “r/science”, on a popular platform, Reddit, in which we investigated the factors contributing to user engagement and perceived effects of science communication from the users’ perspectives. The survey instrument including user engagement scales, perceived effects of science communication, and demographics were distributed among 2000 participants in the r/science Ask Me Anything (AMA) series. We analyzed 146 survey responses using descriptive statistics and ordinal logistic regression. The findings indicated that the participants were generally engaged compared to ones in other studies that used the same user engagement scales and perceived positive effects on science communication, except when it came to building trust. Furthermore, we found that time spent on this particular platform appeared to be the most important factor when it came to positive perceived effects of r/science AMAs. This type of mediated public engagement has been insufficiently investigated, most particularly in terms of the examination of participants’ perspectives. This void is addressed in this study. The findings from the study will also be informative to similar platforms that support mediated public engagement with science.

## Introduction

Effective communication of scientific knowledge encourages the public to take a greater interest in science, value the contributions of scientists, and foster public support for the funding of scientific research [1]. Presently, traditional models of scientific communication are giving way to new types of public engagement with science (PES) as the Internet becomes increasingly ubiquitous. Instead of having scientific findings filtered through traditional mediators (journalists, healthcare professionals, government organizations, etc.), scientists have begun to take advantage of social media in order to communicate directly with the general public [2]. Furthermore, the public now participates in the framing of scientific discoveries and opinions by editing science-related articles on popular websites like Wikipedia [3] and/or by assisting with the collection of data for citizen science projects [4]. National Science Board’s [5] survey found that Americans have become more reliant on the Internet as a source of science and technology information, physically visiting informal science sites, such as zoos and aquariums, less frequently than in the past. Brossard [6] notes that the role of lay participation in online environments has changed the nature of science communication, creating new opportunities for two-way communication.

Yet even though engagement in a robust dialogue with the general public has been encouraged as a means of helping them to understand science [7], many scientists’ online activities (e.g., announcing journal publications) continue to be characterized by the absence of conversation and clarification. The communication tends to be one-way—from the scientist to the public [8]. Studies have challenged the effectiveness of this one-way online science communication with the findings that scientists do little beyond sending out announcements of their publications via social media (e.g., [2, 9]), and that more often than not, there is no action taken to engage and clarify.

In contrast, preliminary research of the online forum Reddit has shown tremendous potential for connecting scientists directly with the general public via social media, especially those interested in science and health-related topics (e.g., [10–12]). Dudo [13] lists the “Science” subreddit as a promising area of research in terms of examining online communication between scientists and the general public. The post-response format of the site and its popularity (the entire Reddit site boasts over 330 million active monthly users) make this platform an ideal proving ground for emerging forms of online and interactive science communication, where scientists have the opportunity to respond, clarify, and engage in a robust dialogue with the public. Still, important questions remain. Specifically, there is a need to determine which factors encourage meaningful online interaction between scientists and the general public.

While PES activities traditionally inhabit physical environments, such as museum exhibits [14] and science festivals [15], this paper focuses on technology mediated PES (mPES) in an online environment. mPES via Reddit, and specifically the sub-Reddit called “r/science,” embodies *broadening participation*—i.e., Reddit is easily accessible (anyone with an Internet connection can participate), and there is no limit to the number of participants. As more people than ever before access scientific information on the Internet [5], there is an increasing need to examine mPES activities so that scientists can continue to improve effective communication with the public. More specifically, we asked the following research questions:

- How engaged are users of r/science Ask Me Anything (AMA)?
  ○ What factors contribute to user engagement?
- What are the perceived effects of r/science AMA?
  ○ What factors contribute to these perceived effects?

## Literature review

Recently, research on technology mPES has prospered. As an example, the journal *Science Communication*’s special issue entitled “Public science in a wired world: How online media are shaping science communication” [9] was devoted to the critical investigation of mPES through a variety of social media. The studies ranged from analyzing Twitter use during a science festival called NanoDay [16] to observing the effectiveness of misinformation correction in a Twitter experiment [17]. Su, et al. [16] found that informational tweets related to NanoDay were largely one-way, although some tweets encouraged participation (e.g., photo sharing of the event) and posted volunteer opportunities which would lead to audience involvement. During their experimental study with misinformation about the Zika virus, Vraga and Bode [17] observed the effectiveness of correcting misinformation online, and found that corrections posted by trustworthy organizations, such as the Centers for Disease Control (CDC), were particularly effective among participants.

Even though it is increasingly common for social media users to distribute scientific information, mPES remains primarily one-way and focuses on the dissemination of knowledge, rather than the cultivation of engaged dialogue. For example, upon analyzing public comments on five YouTube videos created to popularize science, Visbal and Crawford [18] concluded that solid scientific discussion did not occur as much as they had originally anticipated. The percentages of scientific comments that they coded ranged from 2% to 18%. Furthermore, while multidisciplinary content appeared to attract a wider audience (i.e., higher numbers of views and likes), there was a lack of meaningful scientific discourse. Collins, et al. [2], who conducted a survey of over 500 scientists from various disciplines, found that scientists reported the limited use of social media to the public in order to engage them in a discussion of their research. Furthermore, social media, such as Facebook and Twitter, tended to reveal more one-way announcements about scientists’ work than two-way discussions with the general public. Lee, VanDyke, and Cummins reported a similar finding in their study of social media use in National Oceanic and Atmospheric Administration (NOAA): “NOAA is not interacting with publics to create a place for conversation” [19 p.280].

With some forms of mPES, the general public are actively participating in scientific knowledge sharing. For example, parents share tips for dealing with their children’s head lice [20], autism [21], or research findings in science [22]. Furthermore, according to a survey by Collins, et al. [2], a small number of scientists use Reddit—another social media platform which allows two-way online communications. The platform’s science subreddit, in particular, whose post-response format allows anyone to participate, is considered “the world’s largest two-way dialogue between scientists and the public” [11]. It provides learning opportunities for the general public and emerging forms of interactive mPES. Unfortunately, because Reddit’s “upvote” algorithm was altered—leading to a sharp decline in r/science AMA participation—the subreddit moderators decided to cease AMAs in May 2018. To date, little research has been conducted regarding this dynamic mPES r/science AMA community.

### User engagement

As online environments become much more interactive than before, user engagement is considered an important measure for online activities. It serves as a quantifiable indicator of attention and a proxy for the usefulness of an information resource, especially as users, with ever more options, make quick judgments about whether to use one resource over another. In order for there to be a two-way communication, users need to be engaged. As such, we turned to the literature of user engagement which, in online environments, has generally been measured quantitatively based on the number of user interactions, e.g., the number of likes, retweets, clicks, reposts, and comments [23].

O’Brien and Toms [24] proposed different types of user engagement measures using survey questions. Their intention was to establish a cross-implementation scale comparable across different studies of similar or related user experiences. They developed a User Engagement Scale (UES) originally for use in the context of ecommerce sites. Their UES survey consisted of 4 sub-categories: attention, usability, visual appeal, and sense of reward.

This user engagement scale has been employed in a variety of studies to investigate how user engagement relates to, for instance, gamified interfaces [25], videogame play [26], the design of websites for browsing cultural artifacts [27], changing perceptions of persuasive web content [28], and the relationship between user interest and searching behavior [29]. O’Brien [30] identified similar, as well as additional, applications across various domains such as online search, online news, online video, educational applications, haptic applications, consumer engagement, social networking applications, and video game play.

### Effects of science communication

The study of science communication tends to investigate senders rather than receivers of communication. As such, there is rich literature pertaining to what scientists and/or mediators such as journalists try to achieve through science communication.

Dudo and Besley [1] investigated how scientists rate the following five objectives for online science communication. Based on the survey of 390 responses among AAAS members, they found that scientists’ own priorities for science communications were: defending science, informing the public, exciting the public, building trust, and tailoring messages. The first three objectives were very similar to the scientists’ perceptions of their colleagues’ prioritization. Jensen and Holiman studied how the practice of science communication in the UK has changed from the top-down nature of filling in the scientific knowledge deficit (first order) to two-way dialogues and building trust (second order), and eventually educating the public to be “critically informed” [8 p.72] by engaging in scientific discourses (third order). They found that science communication primarily focuses on resolving the knowledge deficit.

## Methods

### Ethics statement

Indiana University’s Institutional Review Board (protocol #1703890480) approved the study. An informed consent form was presented to all participants, and written consent was received once participants agreed to respond to the survey.

### Study site

We selected Reddit, a popular online platform that allows direct interaction between users. Specifically, we examined “AMA” (Ask Me Anything) conversations in the r/science subreddit whose participants are scientists and lay audiences. AMAs allow Reddit users to post questions to a host (for example, an environmental scientist, astrophysicist, or healthcare specialist). Redditors then “upvote” questions, so that the most popular questions can be identified. The host then responds to the initial questions (in theory, those with the most upvotes) as well as follow-up questions. These r/science AMA sessions allowed scientists to interact directly with a public audience in a robust back-and-forth dialogue. As of the data collection in September 2018, the r/science subreddit had 18 million subscribers and was regularly offering AMA sessions on a variety of scientific topics, each hosted by an individual scientist or research team.

### Sample

**Fig 1:**
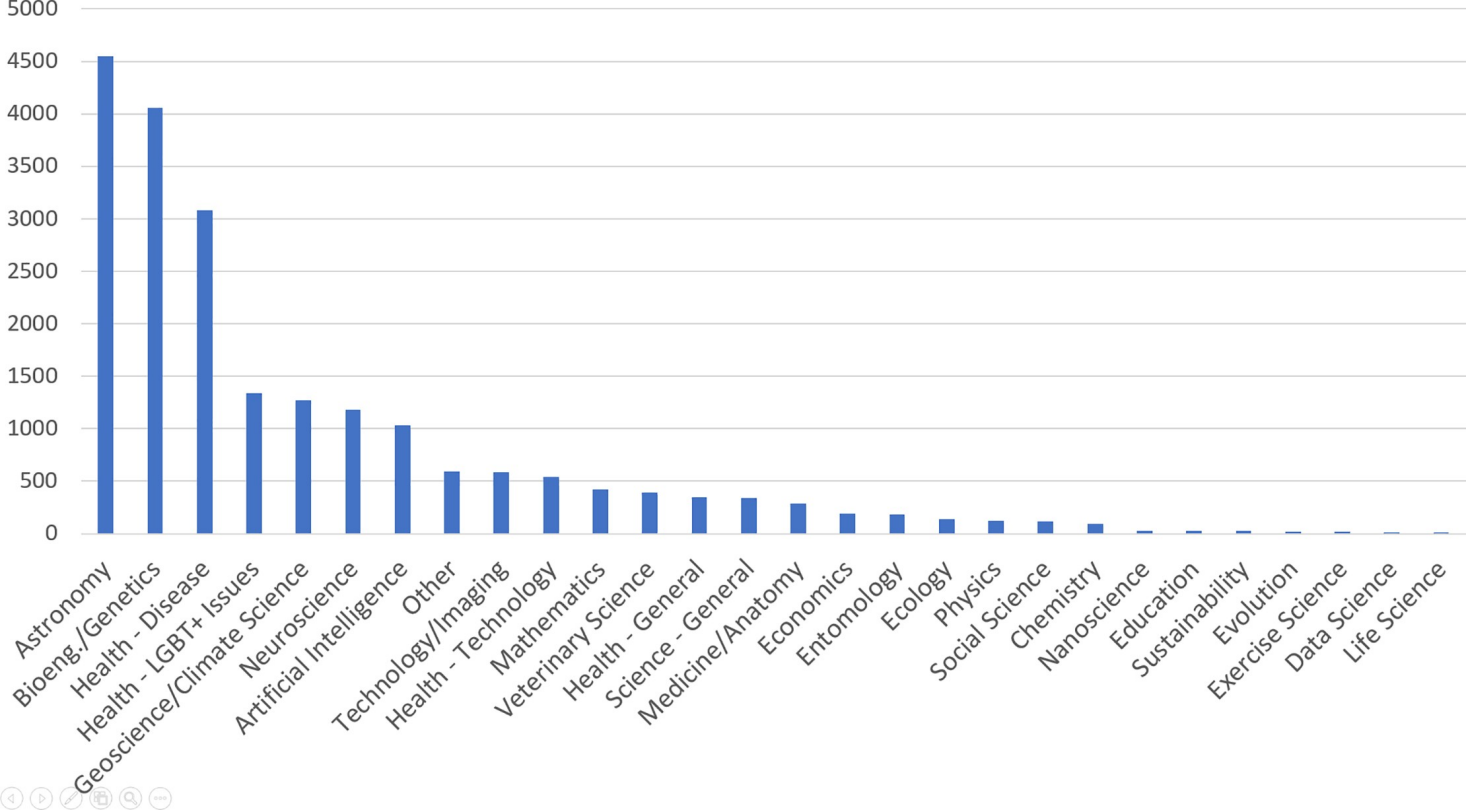
Number of r/science AMAs from each discipline between June 13, 2017 and April 30, 2018 (n = 250).

In order to conduct the survey, we first used the Reddit API to collect information for the most recent 250 posts on r/science tagged as AMAs (the Reddit API was limited to the 250 most recent posts when queried by tag). The API produced 250 AMA sessions posted between June 13^th^, 2017 and April 30^th^, 2018. We then used the Reddit API to collect comments and commenter data for the 250 AMA sessions; a total of 22,337 comments were collected from 13,337 unique reddit user accounts. A unique Reddit user may have more than one account, so an accurate number of individual participants was impossible to gauge. We used the number of unique accounts as a proxy for unique participants. Furthermore, 953 comments whose creators had since deleted their Reddit user accounts were found. These comments were made by an unknown number of unique participants, as the Reddit API does not report the usernames for deleted accounts. AMA sessions were manually coded into scientific disciplines based on their topical tags, the most popular being Health–Disease, Geoscience/Climate Science, and Astronomy. The number of AMAs from each discipline is represented in Fig 1.

Among the user accounts, there were ten organizational AMA accounts, such as AAAS-AMA, HopkinsMedicine_AMA, and NIH_AMA. Because it was obvious that these accounts were used for hosting specific sessions by a group of individuals, they were excluded from the user sample, as were commenters who were current moderators of the r/science subreddit. However, some sampled accounts may have been moderators at some point during the sample timeframe.

Next, we collected data to identify the frequency of user participation for all AMA sessions in our sample. The Reddit API was used to query the comment history of each AMA post individually; each comment, its number of replies, post ID, commenter username, and content were stored for analysis. We found that the participants of r/science AMA had a long-tail distribution in terms of the number of total comments. Among the 23 participants who contributed ten or more comments over the sample timeframe, two made over 200 comments and the remainder made 38 or fewer (it is possible that the top two commenters had been moderators at some point or been involved in a series of long conversation threads). Thus, we decided to sample all participants with two or more comments during the sample time frame (1251) as well as a random sample of 749 participants with a single comment.

**Fig 2:**
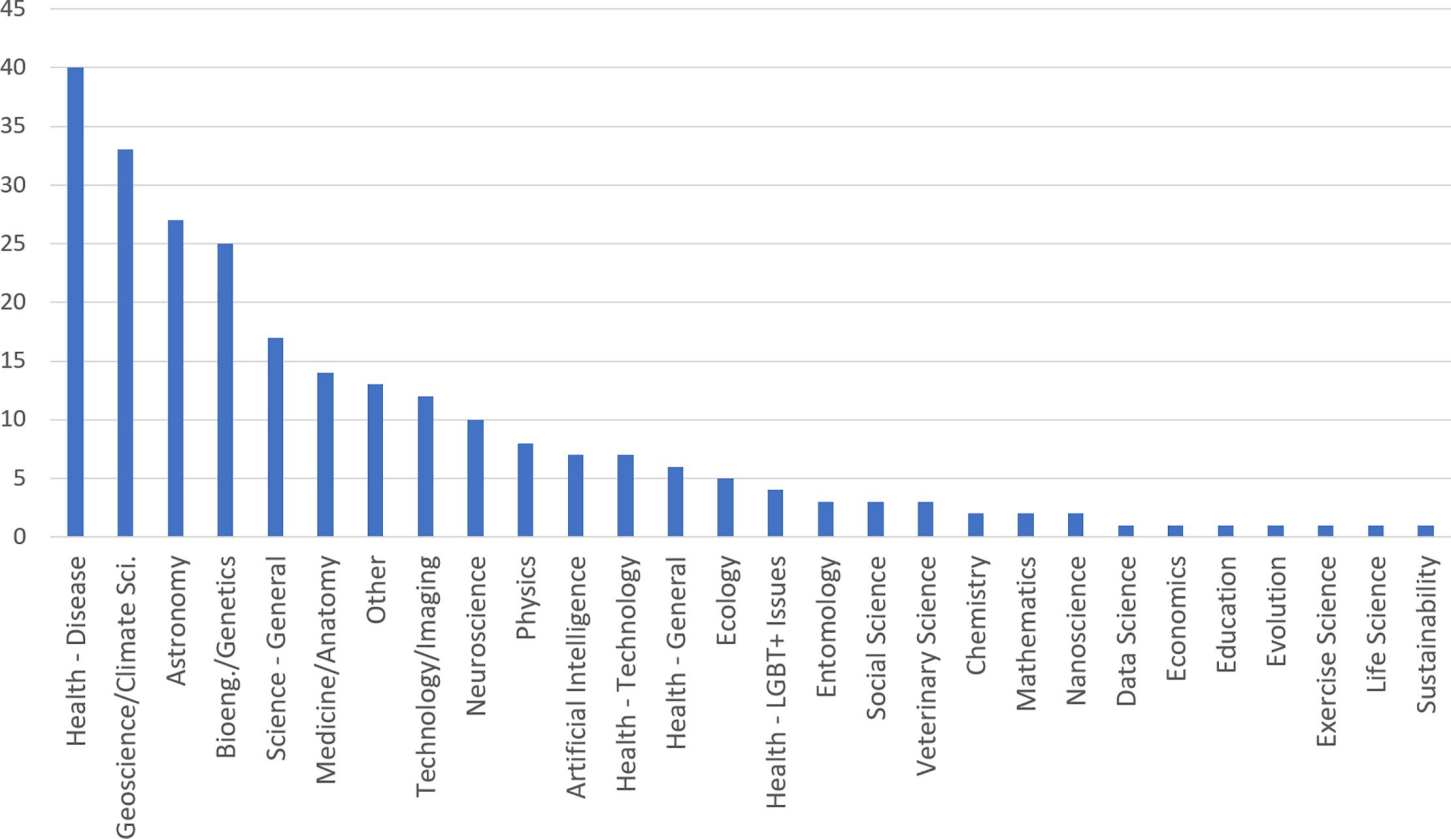
Number of comments by discipline of AMA.

The number of comments for each AMA served as a proxy for the level of overall community participation with the AMA, and by extension, the number of comments served as a proxy for the community’s engagement with each discipline over the sample timeframe. Fig 2 shows the number of comments for AMAs from each discipline in the sample.

The survey was distributed via Reddit message invitations in mid-September 2018 and conducted through Qualtrics. Responses were collected over the course of one month. We used segmented sampling to include a range of participants, from those who had only participated in r/science AMA once, to more frequent participants of r/science AMAs. In total, we distributed the survey to 2000 participants and received 146 responses that completed all questions regarding O’Brien et al [31]’s user engagement scale questions (a 7.3% response rate).

## Measures

Our survey included 33 questions covering three categories: User Engagement, perceived effects of science communication, and demographics.

### User engagement

We used O’Brien et al’s User Engagement Scale (short form) questions in order to gauge user engagement with r/science AMAs. Wiebe, et al. asserted that “self-report measures (usually post hoc) continue to be the most popular measures for characterizing the psychological state of user engagement.” [26 p. 125] The user engagement questions consist of four categories: focused attention (FA); perceived usability (PU); aesthetic appeal (AE), and reward (RW). In keeping with O’Brien et al’s instrument design, we had 12 questions relating to user engagement (see the Supplemental Material S1 for the survey instrument). Among them, three questions for FA asking for users’ concentration levels, based on Flow Theory [26]); three questions for PU asking for users’ negative feelings while interacting with a system; three questions for AE asking for users’ judgement regarding visual attractiveness of a system; three questions for RW asking for users’ satisfaction while using a system). O’Brien et al [31] tested the User Engagement Scale and reported a 95% confidence interval using ω reliability, which is similar to Cronbach α values. The ω values were 0.82, 0.86, 0.84, and 0.81 for FA, PU, AE, and RW respectively. We selected two categories–FA and RW–as our dependent variables because they were more relevant to the questions of engagement. The other two categories (AE and PU) were not the focus of the study as they were related to the platform’s interface features.

### Perceived effects of science communication

From the literature that outlined the goals and expectations for science communicators, we derived the following five goals and expectations indicative of public engagement with science, with the intent to measure:

- Connecting science to everyday lives ([8])
- Making informed decisions using science ([8])
- Being informed ([1, 8])
- Being excited about science ([1, 8])
- Building trust with science ([1, 5, 8])

### Demographics

We also included the following general demographics in the survey: gender, age, education, race, and income. Because mPES does not restrict geographic locations, we wished to identify the locations of the participants. Moreover, since the study site focuses on science discussions, we also wanted to examine whether the participants would consider themselves scientists or not. Additionally, we speculated that the years of Reddit and r/science use might affect the engagement on the site and perceived effects of science communication. Finally, time spent on r/science compared to Reddit as a whole was queried in the survey.

### Analytical strategy

The data related to user engagement was analyzed based on the method suggested by O’Brien et al. [31]. Each of the 6 user engagement survey questions related to focused attention (FA) and reward (RW) was re-coded with a value from 1 to 4, then averaged within each user engagement category for a subscore between 1 and 4. We analyzed the data with linear regression; user engagement was considered a dependent variable. We regressed the FA and RW engagement subscores on users’ demographics and perceived effects of r/science AMAs. The goal was to investigate whether and how these two variables had an impact on the users’ engagement in r/science AMAs. In particular, we investigated perceived reward (RW) and focused attention (FA) for the statistical analysis. If the users perceive positive effects, they are more likely to focus and feel rewarded. Previous studies confirmed that the use of images is a predictor for greater user engagement in space science communication [32] and health communication [33] on social media. However, to our knowledge, the relationship between perceived effects and user engagement have not been studied. Additionally, we analyzed the data with ordinal logistic regression using the perceived effects of r/science AMAs as the dependent variables, and examined whether and how users’ demographics were associated with the perceived effects.

The response rate was relatively low, and some variables contained very few cases in certain categories. As such, we grouped these categories into one for these variables for analysis. As for race, we kept the categories of White and Asian, but grouped the rest into one category called Other. The five-scale perceived effects (strong positive effect, weak positive effect, no effect, weak negative effect, and strong negative effect) were recoded into three categories: strong positive effect, weak positive effect, no effect/negative effect (the final three categories—no effect, weak negative effect, and strong negative effect—were merged into the third category because they comprised a small number of responses).

## Findings

### Descriptive statistics

Table 1 presents descriptive statistics of all of the variables in the study. Regarding the demographics of the respondents, more males (109) than females (36) responded. According to Barthel, Stocking, Holcomb, and Mitchell [34], this number is fairly consistent with the statistics for Reddit as a whole (men being 69% and women being 31%). The authors also found that about a half (43.8%) of Reddit users were younger than 30. Our respondents were relatively older (58% were 18–29 years old and 33% were 30–49 years old) and highly educated—69.2% had at least a college degree. As with the general Reddit user population (White being 70% of the participants), more than half of our survey respondents (66.4%) were white. Unsurprisingly, the respondents were predominantly located in North America (71.9%), as Reddit originated from the U.S. and is primarily an English-speaking platform.

**Table 1:** Descriptive statistics of all variables.

Variables	Mean/Prop	SD	Min	Max	N
**Demographics**					
Sex (Female = 1)	0.25		0	1	145
Education			1	4	135
High school graduate	0.15		0	1	
Two-year associate degree	0.1		0	1	
Four-year college degree	0.39		0	1	
Postgraduate degree	0.36		0	1	
Race			1	4	140
White	0.69		0	1	
Asian	0.12		0	1	
Other	0.19		0	1	
Age			1	5	146
18–24 yrs	0.21		0	1	
25–29 yrs	0.23		0	1	
30–39 yrs	0.29		0	1	
40–49 yrs	0.14		0	1	
50+ yrs	0.13		0	1	
Income			1	4	113
Below $20k	0.21		0	1	
$20k - $50k	0.28		0	1	
$50k - $100k	0.28		0	1	
Above $100k	0.22		0	1	
Location (North America = 1, other = 0)	0.72		0	1	145
Years in r/science			1	5	136
1 year	0.21		0	1	
2 years	0.21		0	1	
3 years	0.18		0	1	
4–5 years	0.2		0	1	
6 years and above	0.2		0	1	
Self-identity as a scientist (Yes = 1)	0.42		0	1	139
Percentage of time on Reddit spent on r/science	17.06	12.54	1	70	141
**User engagement scale**					
Focused attention	2.71	0.85	1	5	146
Reward	3.88	0.62	2	5	146
**Perceived effects of r/science AMAs**					
Connecting with everyday lives			1	5	146
Strong negative effect	0.02		0	1	
Weak negative effect	0.01		0	1	
No effect	0.03		0	1	
Weak positive effect	0.46		0	1	
Strong positive effect	0.48		0	1	
Making informed decisions					146
Strong negative effect	0.02		0	1	
Weak negative effect	0.03		0	1	
No effect	0.14		0	1	
Weak positive effect	0.51		0	1	
Strong positive effect	0.3		0	1	
Being informed					146
Strong negative effect	0.02		0	1	
Weak negative effect	0.01		0	1	
No effect	0.05		0	1	
Weak positive effect	0.44		0	1	
Strong positive effect	0.48		0	1	
Being excited					146
Strong negative effect	0.01		0	1	
Weak negative effect	0		0	1	
No effect	0.13		0	1	
Weak positive effect	0.4		0	1	
Strong positive effect	0.46		0	1	
Building trust					145
Strong negative effect	0.03		0	1	
Weak negative effect	0.02		0	1	
No effect	0.26		0	1	
Weak positive effect	0.37		0	1	
Strong positive effect	0.31		0	1	

### User engagement scales

**Fig 3:**
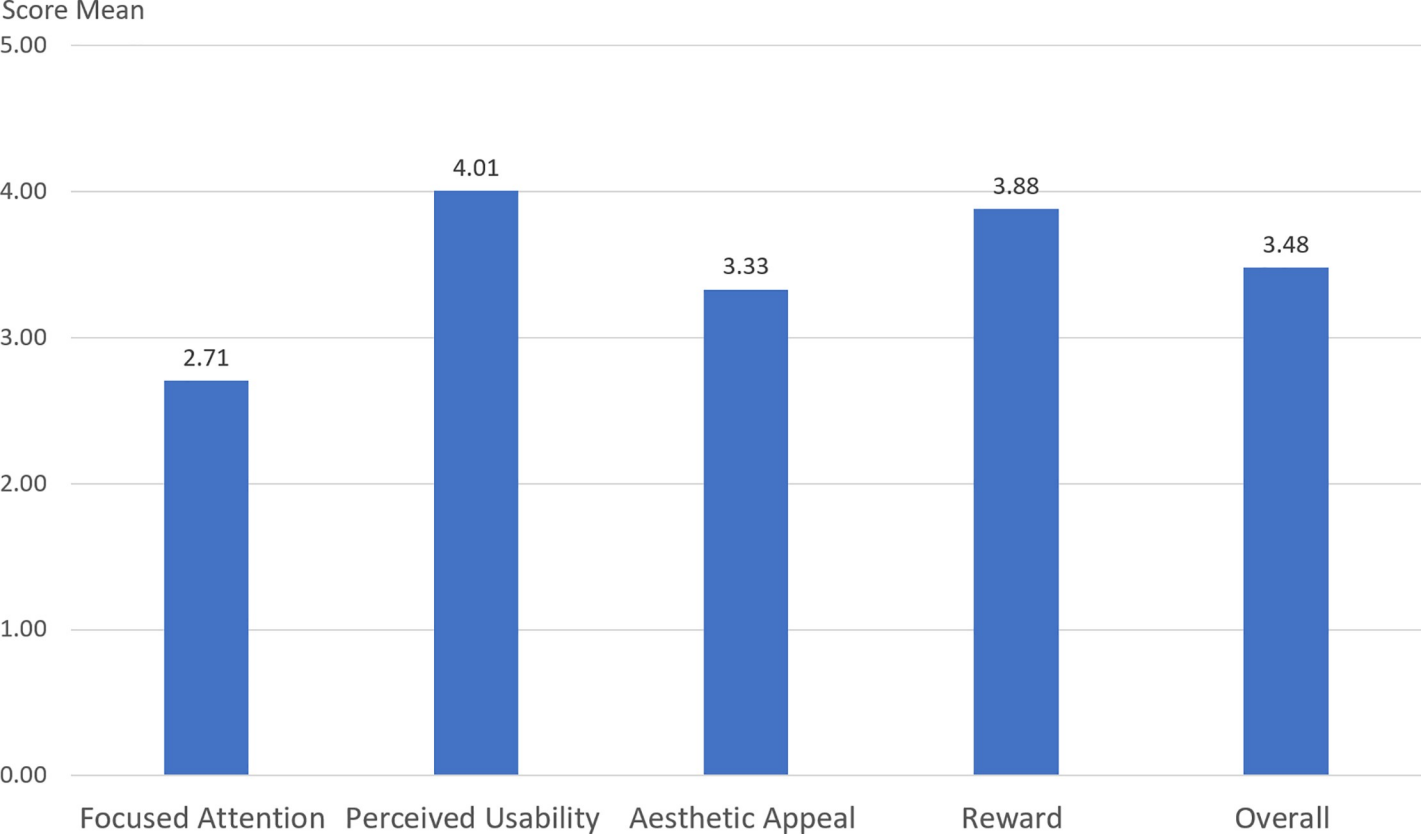
User engagement scale based on O’Brien et al. [31].

Basing off of O’Brien, et al. [31]’s measures, we observed that the participants in r/science AMAs who completed the survey were generally engaged (Fig 3). The overall mean for the user engagement scale was 3.48 out of 5, a result comparable to that found in the experimental conditions of recent studies: 3.77 [35] and 3.8 [36]. It is also similar to the overall mean found in the control or latent conditions of recent studies: 3.61 [29]; 3.325 [36]; 3.5 [25]; and 3.165 [35]. The relative means of the four subscores varied in each of those studies, as did they in ours.

### What factors contribute to user engagement?

We also used the two user engagement measures (FA: focused attention; RW: reward factor) as dependent variables to run the linear regression analysis.

#### Focused attention

Model 1 in Table 2 reports the results of linear regression of users’ demographics and perceived effects on focused attention. We identified that age and scientist identity were negatively associated with focused attention. This means that the older the participants were, and if they self-identified as scientists, the less likely they were to pay attention to r/science AMAs. Another reason for their limited attention is the likelihood that they considered themselves more knowledgeable in general. On the other hand, if the participants had previously spent more time with r/science, they were more likely to have focused attention on r/science AMAs. It is possible that those already familiar with r/science might already be convinced of the value of r/science AMAs, and therefore be more likely to focus their attention on them. Models 2 through 6 added each of the perceived effects of r/Science AMAs, and none of their coefficients were significant, suggesting that perceived effects had no influence on focused attention.

**Table 2:** Results of linear regression of users’ demographics and perceived effects on focused attention (FA).

	Model 1	Model 2	Model 3	Model 4	Model 5	Model 6
Sex (Female = 1)	-0.033	-0.041	-0.037	-0.033	-0.019	-0.044
	(0.181)^a^	(0.185)	(0.182)	(0.182)	(0.182)	(0.183)
Race						
Asian	0.190	0.182	0.175	0.174	0.156	0.191
	(0.240)	(0.244)	(0.241)	(0.243)	(0.243)	(0.241)
Other	0.208	0.209	0.207	0.204	0.201	0.207
	(0.185)	(0.186)	(0.185)	(0.186)	(0.185)	(0.186)
Education	-0.070	-0.070	-0.063	-0.074	-0.066	-0.070
	(0.083)	(0.084)	(0.084)	(0.084)	(0.084)	(0.084)
Age	-0.197**	-0.197**	-0.199**	-0.189**	-0.202**	-0.196**
	(0.067)	(0.067)	(0.067)	(0.069)	(0.067)	(0.067)
Location (North America = 1, other = 0)	0.144	0.147	0.118	0.15	0.151	0.135
	(0.174)	(0.176)	(0.178)	(0.175)	(0.175)	(0.176)
Self-identity as a scientist (Yes = 1)	-0.369*^b^	-0.368*	-0.376*	-0.352*	-0.367*	-0.359*
	(0.161)	(0.162)	(0.161)	(0.164)	(0.161)	(0.163)
Years in r/science	-0.018	-0.019	-0.016	-0.021	-0.019	-0.014
	(0.054)	(0.054)	(0.054)	(0.054)	(0.054)	(0.054)
Percentage of time on Reddit spent on r/science	0.017**	0.017**	0.017**	0.018**	0.018**	0.017**
	(0.006)	(0.006)	(0.006)	(0.006)	(0.006)	(0.006)
**Perceived effects** (positive effect = 1, no/negative effect = 0)						
Connecting with everyday lives		-0.069				
		(0.304)				
Making informed decisions			0.138			
			(0.187)			
Being informed				-0.143		
				(0.265)		
Being excited					-0.182	
					(0.205)	
Building trust						0.077
						(0.155)
Constant	3.196***	3.264***	3.098***	3.312***	3.341***	3.139***
	(0.337)	(0.453)	(0.363)	(0.401)	(0.375)	(0.358)
N	116	116	116	116	116	116
Adjusted R^2^	0.219	0.212	0.216	0.214	0.218	0.214

^a^ Standard errors in parentheses

^b^ * p<0.05, ** p<0.01, *** p<0.001

#### Reward factor

Model 1 in Table 3 reports the results of linear regression of users’ demographics and perceived effects on reward factors. We found that if the participants were female, they were more likely to have high reward subscores. It is possible that, as females are more unlikely than men to pursue science careers [37, 38], fewer women are familiar with and knowledgeable about science than their counterpart, resulting in the tendency of females feeling increasingly rewarded through r/science AMA participation. Another possibility is that females are generally more positive about computer-mediated communication. Chang [39] investigated gender difference in response to conflicting online information about consumer products. She found that women tend to elaborate more on positive consumer reviews of products than men, who tend to elaborate more on negative reviews. She also observed that due to the fact that men generally feel more discomfort with conflicting consumer reviews online, they are less trusting of inconsistent product information. It is possible that this gender difference can be applied to online science information.

**Table 3:** Results of linear regression of users’ demographics and perceived effects on reward (RW).

	Model 1	Model 2	Model 3	Model 4	Model 5	Model 6
Sex (Female = 1)	0.361*^b^	0.453**	0.351*	0.362**	0.326*	0.306*
	(0.140) ^a^	(0.136)	(0.137)	(0.136)	(0.136)	(0.134)
Race						
Asian	-0.109	-0.016	-0.147	-0.047	-0.024	-0.106
	(0.186)	(0.178)	(0.183)	(0.182)	(0.182)	(0.177)
Other	-0.033	-0.038	-0.036	-0.016	-0.015	-0.040
	(0.143)	(0.136)	(0.140)	(0.139)	(0.138)	(0.136)
Education	0.037	0.043	0.053	0.054	0.028	0.036
	(0.065)	(0.061)	(0.064)	(0.063)	(0.062)	(0.062)
Age	-0.078	-0.085	-0.082	-0.107*	-0.066	-0.072
	(0.052)	(0.049)	(0.051)	(0.052)	(0.050)	(0.050)
Location (North America = 1, other = 0)	0.119	0.084	0.057	0.098	0.102	0.073
	(0.135)	(0.129)	(0.135)	(0.132)	(0.131)	(0.129)
Self-identity as a scientist (Yes = 1)	-0.142	-0.155	-0.160	-0.205	-0.147	-0.092
	(0.125)	(0.118)	(0.122)	(0.123)	(0.120)	(0.119)
Years in r/science	-0.050	-0.030	-0.046	-0.039	-0.046	-0.032
	(0.042)	(0.040)	(0.041)	(0.041)	(0.040)	(0.040)
Percentage of time on Reddit spent on r/science	-0.000	-0.002	-0.002	-0.002	-0.003	-0.001
	(0.005)	(0.005)	(0.005)	(0.005)	(0.005)	(0.005)
**Perceived effects** (positive effect = 1, no/negative effect = 0)						
Connecting with everyday lives		0.798***				
		(0.223)				
Making informed decisions			0.339*			
			(0.141)			
Being informed				0.533**		
				(0.199)		
Being excited					0.457**	
					(0.153)	
Building trust						0.394***
						(0.114)
Constant	4.077***	3.287***	3.838***	3.643***	3.714***	3.784***
	(0.261)	(0.331)	(0.274)	(0.301)	(0.280)	(0.263)
N	116	116	116	116	116	116
Adjusted R^2^	0.049	0.145	0.090	0.102	0.115	0.139

^a^ Standard errors in parentheses

^b^ * p<0.05, ** p<0.01, *** p<0.001

Models 2 through 6 added each of the perceived effects, all of which were positively associated with reward factor (p < 0.05). Individuals who believed that r/science AMAs had more positive effects (i.e., connecting science to their everyday lives; making informed decisions using science; being informed; being excited about science; building trust with science) felt that their participation in r/science AMAs was rewarding. This makes sense because both factors (positive effects and rewards) reinforce the other. In other words, people who feel r/science AMAs have more positive effects may attribute value to communicating science with the public via this site, thus generating a sense of achievement and reward.

### Perceived effects based on users’ perspectives

Overall, the participants felt that r/science AMAs had some positive impact for the five perceived effects we surveyed. In particular, over 90% of the respondents perceived that r/science AMAs helped them connect science with their everyday lives (94%) and be informed about science (92%). In terms of negative effects of r/science AMAs for science communication, only a few participants (5%) perceived some negative effects of r/science AMAs upon making informed decisions and building trust for science. More significantly, over a quarter of participants (26%) responded that r/science had no effects with regard to building trust for science, possibly because that was not the focus of r/science AMAs. Generally speaking, it appeared that the purpose of r/science AMAs was to make people more connected to, excited, and informed about science. The founder of r/science AMA, Nathan Allen, commented on the motivation behind creating such a platform in an interview with Chemical & Engineering News, “If scientists are not representing themselves, nobody will represent us for us… We need to show people how great the work we’re doing in chemistry is” [40].

#### What factors contribute to perceived effects of r/science AMAs?

**Fig 4:**
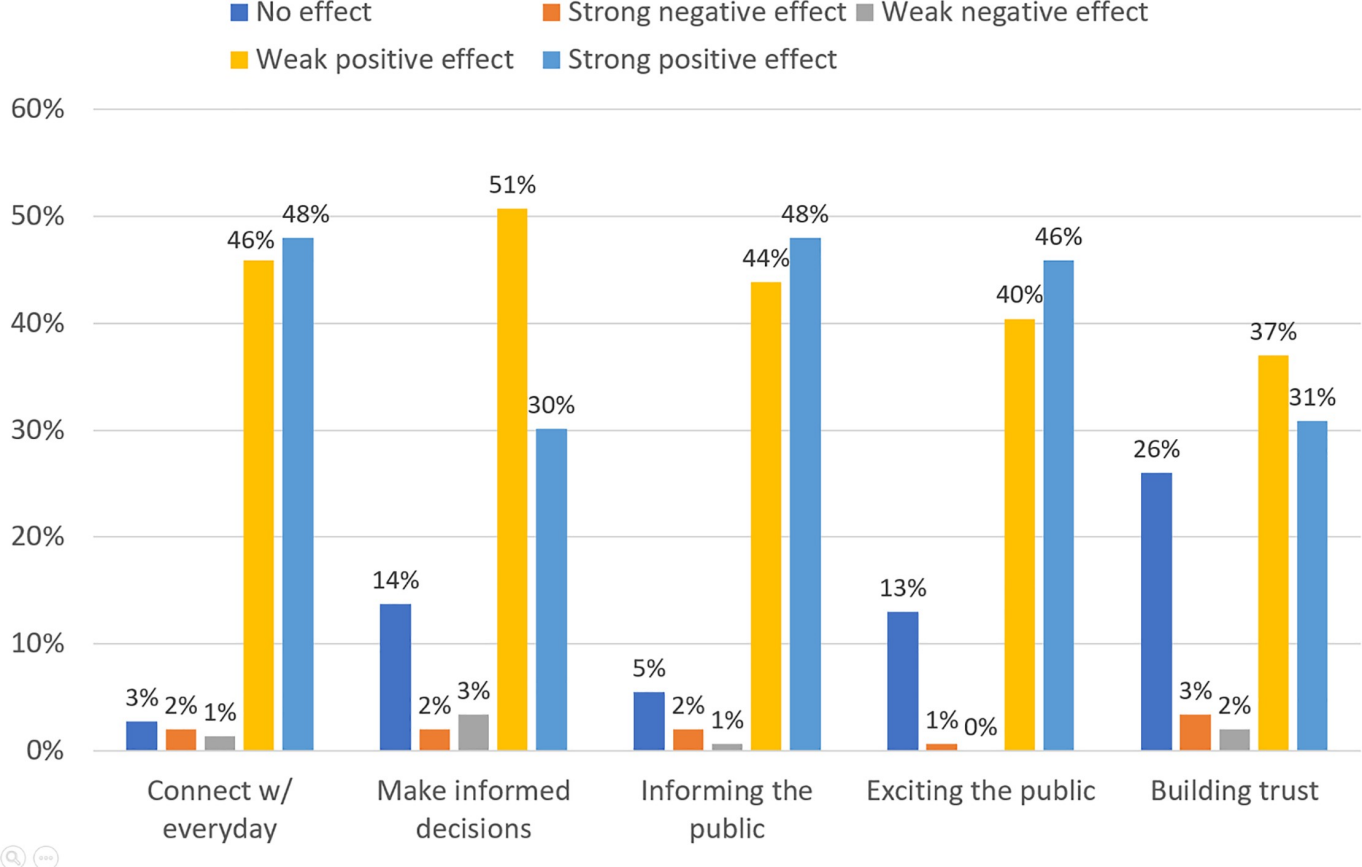
Perceived effects of r/science AMAs.

We used ordinal logistic regression to identify which factors contributed to these perceived effects of r/science AMAs. We documented the five recorded raw response values (Fig 4) and combined them into the three mentioned in the Measures section.

First, we examined factors related to the effect of r/science helping connect science to everyday lives. We found that age was negatively associated with this effect (p < 0.05) (see Model 1 in Table 4). The younger the participants were, the more positive effects of r/science they felt. But the coefficient of age became insignificant after adding years in and time spent on r/science (see Model 2); and the coefficient of time spent on r/science was positively significant (p < 0.05), meaning that time spent on r/science had a mediation effect on the relationship between age and the outcome. That is, the reason younger participants were more likely to think science helps connect science to their everyday lives is because they spent more time on r/science. And the more time the participants spent on r/science, the more positive effect they believed that the r/science had on connecting science to their daily lives.

**Table 4:** Results of ordinal logistic regression of users’ demographics on the perceived effect of helping connect science to their everyday lives.

	All		Scientists		Non-scientists	
	Model 1	Model 2	Model 3	Model 4	Model 5	Model 6
Sex (Female = 1)	0.532	0.104	0.199	-0.238	1.075	0.869
	(0.461)^a^	(0.506)	(0.717)	(0.780)	(0.684)	(0.776)
Race						
Asian	0.166	-0.065	1.382	1.488	-0.619	-1.740
	(0.597)	(0.685)	(1.072)	(1.095)	(0.764)	(1.028)
Other	-0.225	-0.148	0.176	0.562	-0.621	-0.733
	(0.464)	(0.503)	(0.742)	(0.786)	(0.654)	(0.726)
Education	-0.139	-0.003	-1.520*^b^	-1.244	0.164	0.291
	(0.212)	(0.228)	(0.635)	(0.685)	(0.244)	(0.278)
Age	-0.398*	-0.351	-0.358	-0.323	-0.461*	-0.410
	(0.167)	(0.179)	(0.279)	(0.307)	(0.223)	(0.245)
Location (North America = 1, other = 0)	0.278	0.278	-0.724	-0.485	0.163	0.462
	(0.475)	(0.475)	(0.756)	(0.779)	(0.598)	(0.691)
Self-identity as a scientist (yes = 1)	-0.490	-0.609	-	-	-	-
	(0.412)	(0.444)	-	-	-	-
Years in r/science		-0.136		0.016		-0.309
		(0.147)		(0.250)		(0.200)
Percentage of time on Reddit spent on r/science		0.042*		0.020		0.051*
		(0.018)		(0.034)		(0.025)
N	125	116	51	49	74	67

^a^ Standard errors in parentheses

^b^ * p<0.05, ** p<0.01, *** p<0.001

We observed a few things upon separating the participants who self-identified as scientists from those who self-identified as non-scientists. First, the coefficient of education was significantly negative (p < 0.05) (see Model 3), meaning that the higher the education the participants obtained, the fewer positive effects they felt. However, this education effect was no longer relevant after adding years in r/science and time spent on r/science (see Model 4). This suggests that the education effect may be due to the fact that the more educated the participants were, the less time they spent on r/science, thus adding to the unlikeliness of them feeling positive effects of connecting science to their daily lives. Second, the coefficient of age was significantly negative (p < 0.05) for non-scientists (see Model 5), but such an effect became insignificant after adding time spent on r/science, whose coefficient was significantly positive (see Model 6). This was similar to the results of Models 1 and 2.

Second, we analyzed factors related to the effect of r/science on helping individuals make informed decisions based on science. We found that the coefficient of female was significantly positive (p < 0.05) (see Model 1 in Table 5), but this coefficient became insignificant after adding time spent on r/science, whose coefficient was significantly positive (see Model 2). This suggests that the fact that females (compared with males) tended to perceive greater helpfulness of r/science in making informed decisions was due to the fact that females spent more time on r/science. The more time the participants spent on r/science, the more positively effective they also believed it was in helping individuals make informed decisions based on science. When separating the scientists from non-scientists, we found that only the coefficient of time spent on r/science was significantly positive for non-scientists (see Model 6). That is, the more time spent on r/science, the more positive effects the non-scientists felt r/science had on making informed decisions.

**Table 5:** Results of ordinal logistic regression of users’ demographics on the perceived effect of r/science helping individuals make informed decisions based on science.

	All		Scientists		Non-scientists	
	Model 1	Model 2	Model 3	Model 4	Model 5	Model 6
Sex (Female = 1)	1.025*^b^	0.628	1.024	0.555	1.141	1.007
	(0.440)^a^	(0.484)	(0.675)	(0.739)	(0.612)	(0.681)
Race						
Asian	0.664	0.552	0.828	0.899	0.515	0.192
	(0.576)	(0.642)	(0.938)	(0.964)	(0.747)	(0.918)
Other	-0.725	-0.821	-0.802	-0.747	-0.701	-0.884
	(0.446)	(0.495)	(0.690)	(0.727)	(0.604)	(0.700)
Education	-0.402	-0.314	-0.814	-0.497	-0.280	-0.275
	(0.207)	(0.225)	(0.437)	(0.482)	(0.238)	(0.270)
Age	-0.085	-0.029	-0.081	-0.008	-0.114	-0.101
	(0.162)	(0.177)	(0.270)	(0.297)	(0.208)	(0.235)
Location (North America = 1, other = 0)	0.301	0.760	-0.255	0.068	0.548	1.293
	(0.437)	(0.474)	(0.719)	(0.746)	(0.583)	(0.683)
Self-identity as a scientist (yes = 1)	0.128	0.143	-	-	-	-
	(0.405)	(0.436)	-	-	-	-
Years in r/science		-0.113		-0.058		-0.173
		(0.145)		(0.235)		(0.193)
Percentage of time on Reddit spent on r/science		0.042*		0.038		0.058*
		(0.018)		(0.031)		(0.024)
N	125	116	51	49	74	67

^a^ Standard errors in parentheses

^b^ * p<0.05, ** p<0.01, *** p<0.001

Third, as for the influence of r/science on users who want to be informed, we found that the coefficient of race (i.e., the category of other) was significantly negative (p < 0.05) (see Model 1 in Table 6). In other words, compared with White, other races (including Asian) were less likely to believe that r/science had a positive effect on being helpful with regard to informing participants. But the coefficient of race was no longer relevant after adding time spent on r/science, whose coefficient was significantly positive (see Model 2). That is, the more time the participants spent on r/science, the more a positive effect they believed r/science had. Moreover, the racial difference was likely due to the fact that other racial categories spent less time on r/science.

**Table 6:** Results of ordinal logistic regression of users’ demographics on the perceived effect of r/science being helpful with regard to informing participants.

	All		Scientists		Non-scientists	
	Model 1	Model 2	Model 3	Model 4	Model 5	Model 6
Sex (Female = 1)	0.616	0.276	1.070	0.747	0.759	0.604
	(0.442)^a^	(0.476)	(0.724)	(0.784)	(0.644)	(0.707)
Race						
Asian	0.185	-0.037	1.054	1.028	-0.263	-0.935
	(0.585)	(0.662)	(1.030)	(1.031)	(0.740)	(0.933)
Other	-0.931*^b^	-0.822	-0.733	-0.337	-1.209	-1.293
	(0.454)	(0.481)	(0.723)	(0.752)	(0.627)	(0.689)
Education	-0.030	0.091	-0.572	-0.512	0.061	0.171
	(0.206)	(0.221)	(0.468)	(0.523)	(0.241)	(0.268)
Age	-0.155	-0.088	-0.294	-0.221	-0.144	-0.098
	(0.162)	(0.174)	(0.276)	(0.302)	(0.219)	(0.241)
Location (North America = 1, other = 0)	0.149	0.421	-1.317	-1.198	-0.752	1.201
	(0.430)	(0.461)	(0.772)	(0.785)	(0.575)	(0.671)
Self-identity as a scientist (yes = 1)	-0.238	-0.286	-	-	-	-
	(0.405)	(0.432)	-	-	-	-
Years in r/science		-0.176		-0.197		-0.243
		(0.141)		(0.240)		(0.188)
Percentage of time on Reddit spent on r/science		0.042*		0.003		0.034
		(0.018)		(0.032)		(0.022)
N	125	116	51	49	74	67

^a^ Standard errors in parentheses

^b^ * p<0.05, ** p<0.01, *** p<0.001

Fourth, the participants who spent more time on r/science reported their belief that r/science had positive effects on exciting the public about science (see models 2, 4, and 6 in Table 7). It is encouraging that users who spent the most time on r/science, and who presumably knew most about its content and patterns, believed that r/science is capable of significantly impacting public excitement about science and scientific topics—as a lack of excitement would surely be a strong barrier to public engagement.

**Table 7:** Results of ordinal logistic regression of users’ demographics on the perceived effect of r/science exciting the public about science.

	All		Scientists		Non-scientists	
	Model 1	Model 2	Model 3	Model 4	Model 5	Model 6
Sex (Female = 1)	0.467	0.176	0.889	0.407	0.130	-0.180
	(0.426)^a^	(0.466)	(0.663)	(0.724)	(0.586)	(0.645)
Race						
Asian	-0.490	-1.018	-0.083	-0.260	-0.644	-1.501
	(0.545)	(0.620)	(0.931)	(0.944)	(0.692)	(0.899)
Other	-0.619	-0.829	-0.834	-1.255	-0.373	-0.524
	(0.433)	(0.461)	(0.651)	(0.708)	(0.612)	(0.655)
Education	0.056	0.116	0.237	0.705	0.038	0.003
	(0.201)	(0.217)	(0.409)	(0.484)	(0.236)	(0.257)
Age	-0.089	-0.095	-0.090	-0.002	-0.167	-0.119
	(0.161)	(0.173)	(0.267)	(0.289)	(0.211)	(0.229)
Location (North America = 1, other = 0)	0.097	0.052	0.482	0.693	-0.013	-0.206
	(0.407)	(0.439)	(0.688)	(0.730)	(0.550)	(0.634)
Self-identity as a scientist (yes = 1)	-0.293	-0.440	-	-	-	-
	(0.392)	(0.417)	-	-	-	-
Years in r/science		-0.081		-0.164		-0.064
		(0.135)		(0.224)		(0.178)
Percentage of time on Reddit spent on r/science		0.043*^b^		0.065*		0.049*
		(0.017)		(0.032)		(0.023)
N	125	116	51	49	74	67

^a^ Standard errors in parentheses

^b^ * p<0.05, ** p<0.01, *** p<0.001

Finally, we investigated if any of the factors were related to the belief that r/science helped build trust for science. We found that if participants were female, they were more likely to believe that r/science AMAs helped build trust for science (see Model 1 in Table 8). But the gender difference was no longer significant after adding time spent on r/science (see Model 2). It is possible that females participated more in r/science and thus were more likely to feel positive effects of building trust. Again, when we separated self-identifying from non-identifying scientists, the results showed that being female had a significant positive statistical difference only if the participants self-identified as non-scientists (see Models 5 and 6). Additionally, highly educated individuals were less likely to consider that r/science had positive effects on building trust for science only if they self-identified as scientists (see Model 3). But the education effect became insignificant after factoring in time spent on r/science (see Model 4). It is probable that highly educated individuals participated less in r/science and thus were less likely to feel positive effects of building trust.

**Table 8:** Results of ordinal logistic regression of users’ demographics on the perceived effect of r/science in helping built trust for science.

	All		Scientists		Non-scientists	
	Model 1	Model 2	Model 3	Model 4	Model 5	Model 6
Sex (Female = 1)	0.998*^b^	0.739	0.929	0.389	1.353*	1.378*
	(0.417)^a^	(0.460)	(0.629)	(0.696)	(0.610)	(0.673)
Race						
Asian	0.616	0.463	1.439	1.430	0.047	-0.033
	(0.564)	(0.626)	(1.012)	(1.030)	(0.728)	(0.892)
Other	-0.282	-0.206	-0.329	-0.231	-0.320	-0.245
	(0.427)	(0.463)	(0.659)	(0.700)	(0.596)	(0.653)
Education	-0.122	0.069	-0.843*	-0.578	0.137	0.388
	(0.193)	(0.204)	(0.417)	(0.465)	(0.223)	(0.241)
Age	-0.049	-0.033	0.348	0.473	-0.300	-0.314
	(0.156)	(0.166)	(0.257)	(0.283)	(0.211)	(0.228)
Location (North America = 1, other = 0)	0.267	0.532	0.176	0.476	0.309	0.708
	(0.415)	(0.442)	(0.687)	(0.732)	(0.551)	(0.627)
Self-identity as a scientist (yes = 1)	-0.466	-0.704	-	-	-	-
	(0.383)	(0.406)	-	-	-	-
Years in r/science		-0.175		-0.222		-0.176
		(0.135)		(0.225)		(0.178)
Percentage of time on Reddit spent on r/science		0.043		0.039		0.000
		(0.017)		(0.031)		(0.021)
N	125	116	51	49	74	67

^a^ Standard errors in parentheses

^b^ * p<0.05, ** p<0.01, *** p<0.001

## Discussions & conclusions

Overall, respondents had UES scores similar to those found in other recent studies [e.g., 25, 29, 35, 36]. This suggests that r/science AMAs were generally engaging, though not to an extraordinary extent. Differences between groups, particularly between males and females, scientists and non-scientists, and long-time users and non-long-time users, indicate that engagement in r/science AMAs was not universal, but dependent on several factors—the most significant of which seems to be the duration of one’s participation in the community.

The participants who reported that r/science AMAs had positive effects in communicating science for all five aspects tended to feel an increasing sense of reward by participating in r/science AMAs. This makes sense, but is of little use to those who already perceive great value in engaging with science. In other words, the participants who did not experience these positive effects of science communication responded that they did not experience any reward effect. In fact, the latter group is the demographic on which proponents of public engagement with science should be more focused.

No effects were found regarding whether or not the participants believed in r/science AMAs’ positive influence on communicating science for focused attention. Rather, we observed that the older participants and those who self-identified as scientists were less likely to focus their attention on the AMAs. It is possible that these populations may have less trust in social media and are, as a result, more critical about the effects of r/science AMAs. Finally, the participants who spent more time on r/science AMAs were more likely to focus their attention on them. It is possible that their previous experiences helped them understand the platform better and develop more realistic expectations for these sessions. And perhaps as the participants paid more attention to r/science, they spent more time on it. Thus, the direction of the relationship is reversable.

Similarly, time spent on r/science AMAs seems to be the most important factor on the perceived effects of r/science. This means that we need to make sure that online mPES provide a space in which people are more likely to spend time and to which they will want to return again and again. The positive scores of User Engagement measures for the Reddit site were encouraging. If we want to host a similar program, we ought to make sure that the platform’s design is as enjoyable and user-friendly as Reddit. In addition, the demographic differences we observed in perceived effects were most likely attributed to the differences in time spent on r/science AMAs among these social groups. Younger users, females, Whites, Asians, and less educated users perceived more positive effects from the site because they were more likely to spend time on r/science AMAs. This is an encouraging finding because younger, female, and less educated populations appear to have found this type of venue for science communication useful. Since the time spent is a major factor for positive perceived effects of r/science, it is important to reach out to other populations including older users, males, and other races, and make sure they too are spending time with r/science AMAs. It is possible that this population may not be aware of r/science AMAs and/or their usefulness, or that they are simply disinterested in r/science AMAs.

As r/science ceases to exist, investing in similar mPES venues that incorporate two-way communications between scientists and laypeople, such as r/askscience, Reddit’s general r/AMAs and r/IAmA (i.e., I am a …) featuring scientists, will be beneficial. Even though r/science no longer hosts science specific AMAs, they do post the announcements of AMAs with scientists on r/science. As this type of AMA has been successful, scientists and scientific organizations should consider developing similar setups on popular social media platforms, such as Instagram and Facebook. Live Facebook Q&A sessions have been used to ask experts questions. The Chinese platform called Zhihu Live is designed to facilitate online live Q&A sessions with experts in various fields, not just science [41]. These could both be more systematically organized. It would be beneficial for social scientists who specialize in science communication to be involved to address some overlooked issues such as how to build trust for science among the general public.

Despite these intriguing findings, some limitations for the study need to be considered. First, we had difficult time recruiting the participants, which resulted in the low response rate. In addition, because the survey results were based on those willing to participate in the survey, there was a selection bias. Due to some complication of being able to contact the r/science AMA users, the study suffered from low participation. Furthermore, the study was situated in the subreddit dedicated to the topic of science (i.e., r/science). This space attracts those who are already interested in science. Finally, the study measured User Engagement using the scale some time after the users interacted with the system, not during or immediately afterwards. To mitigate the problem, a survey question had participants answer a r/science AMA-specific question regarding their personal experience with the AMA. Nevertheless, some users may not have remembered exactly how engaged they were while interacting with the system. With these constraints in mind, the generalization of the study is limited. At the same time, previous research has not collected much data from users’ perspective in two-way mPES environments. Our study provides a window to shed light on this phenomenon, and we hope this investigation is just the start.

Future research can expand this current study in several ways. First, even though we examined variation based on respondent identity, we did not investigate the type of subreddit in which more users were active. Incorporating the topics into analysis will likely yield informative results. Second, the study found that the participants did not feel that r/science AMA helped build trust in science despite its popularity. Since gaining trust among the general public is one of the important aspects of science communication, future research can investigate how mPES will facilitate trust building among the general public. Third, while we used a specific platform of reddit for the study, future research can be expanded to other platforms, such as Facebook live Q&A sessions, with an emphasis on a wider population and their public engagement with science online.

This type of mPES is emerging from other platforms, e.g., science blogs, Twitter, and Facebook [9]. As people spend more time online to find scientific information and less time on physical engagement with science activities [5], further research is sought in order for mPES to be more engaging and reach wider populations.

## Supporting information

S1 File Survey instrument for participants in science subreddit AMA. (DOCX)

## References

[1] Dudo A, Besley JC. Scientists’ prioritization of communication objectives for public engagement. PLoS ONE. 2016;11(2):e0148867. 10.1371/journal.pone.0148867 26913869 PMC4767388

[2] Collins K, Shiffman D, Rock J. How are scientists using social media in the workplace? PLoS ONE. 2016;11(10):e0162680. 10.1371/journal.pone.0162680 27732598 PMC5061391

[3] Hara N, Sanfilippo MR. Analysis of roles in engaging contentious online discussions in science. Journal of the Association for Information Science & Technology. 2017;68(8): 1953–1966.

[4] Crowston K, Mitchell EM, Østerlund C. Coordinating advanced crowd work: extending citizen science. Hawaii International Conference on System Sciences. 2018.

[5] National Science Board. Science & engineering indicators. National Science Board. 2018. Available from: https://www.nsf.gov/statistics/2018/nsb20181/

[6] Brossard D. New media landscapes and the science information consumer. Proceedings of the National Academy of Science U S A. 2013;110(3):14096–14101. 10.1073/pnas.1212744110 23940316 PMC3752175

[7] Stilgoe J, Lock SJ, Wilsdon J. Why should we promote public engagement with science? Public Understanding of Science. 2014;23(1):4–15. 10.1177/0963662513518154 24434705 PMC5753839

[8] Jensen E, Holliman R. Norms and values in UK science engagement practice. International Journal of Science Education. 2016;Part B;6(1):68–88.

[9] Davies SR, & Hara N, editors. Special issue: public science in a wired world: how online media are changing science communication. Science Communication. 2017;39(5):563–684.

[10] Lee D. The new Reddit journal of science. Impress Magazine [Internet]. 2015 3 [cited 2018 Aug 4]. Available from: http://www.immpressmagazine.com/the-new-reddit-journal-of-science/

[11] Owens S. The world’s largest 2-way dialogue between scientists and the public. Scientific American. 2014. https://www.scientificamerican.com/article/the-world-s-largest-2-way-dialogue-between-scientists-and-the-public/

[12] Fontaine G, Lavallée A, Maheu-Cadotte MA, Bouix-Picasso J, Bourbonnais A. Health science communication strategies used by researchers with the public in the digital and social media ecosystem: a systematic scoping review protocol. BMJ Open. 2018;8(1): e019833. 10.1136/bmjopen-2017-019833 29382682 PMC5829594

[13] Dudo A. Scientists, the media, and the public communication of science. Social Compass. 2015;9(9):761–775.

[14] Davies SR. Doing dialogue: genre and flexibility in public engagement with science. Science as Culture. 2009;18(4):397–416.

[15] Jensen E, Buckley N. Why people attend science festivals: interests, motivations and self-reported benefits of public engagement with research. Public Understanding of Science. 2014;23(5):557–573. 10.1177/0963662512458624 25414922

[16] Su LYF, Scheufele DA, Bell L, Brossard D, Xenos MA. Information-sharing and community-building: exploring the use of Twitter in science public relations. Science Communication. 2017;39(5):569–597.

[17] Vraga EK, Bode L. Using expert sources to correct health misinformation in social media. Science Communication. 2017;39(5):621–645.

[18] Visbal JLC, Crawford L. Science popularization videos by independent YouTube creators and user’s appropriation strategies: qualitative analysis of user comments. 9th International Conference on Education and New Learning Technologies. 2017:1546–1554.

[19] Lee NM, VanDyke MS, Cummins RG. A missed opportunity?: NOAA’s use of social media to communicate climate science. Environmental Communication. 2017;12(2).

[20] Hine C. Headlice eradication as everyday engagement with science: an analysis of online parenting discussions. Public Understanding of Science. 2014;23(5):574–591. 10.1177/0963662512453419 25414923

[21] Kane GC, Johnson J, Majchrzak A. Emergent life cycle: the tension between knowledge change and knowledge retention in open online coproduction communities. Management Science. 2014;60(12):3026–3048.

[22] Milkman KL, Berger J. The science of sharing and the sharing of science. Proceedings of the National Academy of Sciences of the United States of America. 2014;111(4):13642–13649. 10.1073/pnas.1317511111 25225360 PMC4183177

[23] Lalmas M, O’Brien H, Yom-Tov E. Measuring user engagement. Morgan & Claypool Publishers. 2014.

[24] O’Brien HL, Toms EG. The development and evaluation of a survey to measure user engagement in ecommerce environments. Journal of the American Society for Information Science & Technology. 2010;61(1);50–69. 10.1002/asi.21229

[25] Bertholdo APO, Melo C, Rozestraten AS, Gerosa MA, O’Brien H. User engagement in an open collaboration community after the insertion of a game design element: an online field experiment. Twenty-fourth Americas Conference on Information Systems, New Orleans. 2018.

[26] Wiebe EN, Lamb A, Hardy M, Sharek D. Measuring engagement in video game-based environments: investigation of the User Engagement Scale. Computers in Human Behavior. 2014;32:123–132.

[27] Speakman R, Hall MM, Walsh D. User engagement with generous interfaces for digital cultural heritage. International Conference on Theory and Practice of Digital Libraries. 2018;186–191.

[28] Oh J, Sundar SS. What happens when you click and drag: unpacking the relationship between on-screen interaction and user engagement with an anti-smoking website. Health Communication. 2020;35(3):269–280. 10.1080/10410236.2018.1560578 30618306

[29] O’Brien HL, Arguello J, Capra R. An empirical study of interest, task complexity, and search behaviour on user engagement. Information Processing & Management. 2020;57(3):102226.

[30] O’Brien HL. Why engagement matters. Springer, Cham: Switzerland; c2016. Chapter 2, Translating theory into methodological practice; p. 27–52.

[31] O’Brien HL, Cairns P, Hall M. A practical approach to measuring user engagement with the refined user engagement scale (UES) and new UES short form. International Journal of Human-Computer Studies. (2018);112:28–39. 10.1016/j.ijhcs.2018.01.004

[32] Hwong Y, Oliver C, Van Kranendonk M, Sammut C, Seroussi Y. What makes you tick? The psychology of social media engagement in space science communication. Computers in Human Behavior. 2017;68:480–492.

[33] Rus HM, Cameron LD. Health communication in social media: message features predicting user engagement on diabetes-related Facebook pages. Annals of Behavioral Medicine. 2016;50(5):678–689. 10.1007/s12160-016-9793-9 27059761

[34] Barthel M, Stocking G, Holcomb J, Mitchell A. Nearly eight-in-ten reddit users get news on the site. Pew Research Center [Internet]. 2016 2 [cited 2020 Mar 30]. Available from https://www.pewresearch.org/wp-content/uploads/sites/8/2016/02/PJ_2016.02.25_Reddit_FINAL.pdf

[35] Limerick H, Hayden R, Beattie D, Georgiou O, Müller J. User engagement for mid-air haptic interactions with digital signage. Proceedings of the 8th ACM International Symposium on Pervasive Displays. 2019:1–7.

[36] Nguyen D, Meixner G. Gamified augmented reality training for an assembly task: a study about user engagement. 019 Federated Conference on Computer Science and Information Systems (FedCSIS). 2019:901–904:IEEE.

[37] Castillo R, Grazzi M, Tacsir E. Women in science and technology: what does the literature say? Inter-American Development Bank. 2014. Technical Note No. IDB-TN-637.

[38] Makarova E, Aeschlimann B, Herzog W. The gender gap in STEM fields: the impact of the gender stereotype of math and science on secondary students’ career aspirations. Frontiers in Education. 2019;4(60). https://www.frontiersin.org/articles/10.3389/feduc.2019.00060/full

[39] Chang C. Responses to conflicting information in computer-mediated communication: gender difference as an example. New Media & Society. 2016;18(1) 5–24.

[40] Widener A. Head moderator of Reddit’s science community says chemists need to be more active online. Chemical & Engineering News. 2017;95(46). https://cen.acs.org/articles/95/i46/Head-moderator-Reddit-science-community-says-chemists-need-to-be-more-active-online.html

[41] Tang Y, Tian H, Hara N. Mobile-based synchronous Q&A for knowledge sharing: a case study of Zhihu Live. Proceedings of ASIS&T Regional Meetings—the Asia-Pacific Regional Conference. 2019:22–31.

