# Supplementary material for: Investigating mediated public engagement with science on the “science” subreddit: From the participants’ perspective
Source: PLoS One. 2021 Apr 28;16(4):e0249181. doi: 10.1371/journal.pone.0249181 (PMC8081246; doi:10.1371/journal.pone.0249181)
Supplement: S1 File — (DOCX) [file pone.0249181.s001.docx]

**S1 File.**

Reddit r/Science AMA Commenters Survey Instrument

***Establishing recent or general***

1. Do you clearly recall your most recent experience with an r/Science AMA?
   1. Yes
      1. If Yes is selected, what was the subject of your most recent r/Science AMA?
   2. No

***Engagement questions:***

1. With regard to my participation in ${e://Field/AMA-description}...

|  | Strongly disagree | Disagree | Neither agree nor disagree | Agree | Strongly agree |
| --- | --- | --- | --- | --- | --- |
| I lost myself in this experience. |  |  |  |  |  |
| The time I spent during this experience just slipped away. |  |  |  |  |  |
| I was absorbed in this experience. |  |  |  |  |  |
| I felt frustrated during this experience. |  |  |  |  |  |
| I found the r/Science AMA format confusing to use. |  |  |  |  |  |
| Participating in the r/Science AMA was taxing. |  |  |  |  |  |
| The r/Science AMA format was attractive. |  |  |  |  |  |
| The r/Science AMA format was aesthetically appealing. |  |  |  |  |  |
| The r/Science AMA appealed to my senses. |  |  |  |  |  |
| Participating in the r/Science AMA was worthwhile. |  |  |  |  |  |
| My experience was rewarding. |  |  |  |  |  |
| I felt interested in this experience. |  |  |  |  |  |

***General Reddit and r/Science***

1. Approximately how many years have you been a Reddit user?
2. What are the 3 subreddits with which you engage most?
3. In the past year, which of the following activities have you done on Reddit? (Check all that apply)
   1. Submitted a link
   2. Submitted a text post
   3. Upvoted and/or downvoted posts
   4. Commented on posts
   5. Viewed posts
   6. Participated in an AMA on r/Science
   7. Hosted an AMA on r/Science
   8. Participated in an AMA outside of r/Science
   9. Hosted an AMA outside of r/SCience
4. Approximately how many years have you been a participant in r/Science?
5. In a typical week or month, approximately what percent of your time on Reddit is spent on r/Science or engaging with r/Science content?
6. Professionally, do you identify as a scientist?
   1. Yes
   2. No
7. Is your interest in r/Science personal or professional?
   1. Purely personal (curiosity/interest in science)
   2. More personal than professional
   3. Both personal and professional
   4. More professional than personal
   5. Purely professional (networking/keeping up with new research)
8. What effect do you believe r/Science AMAs have on the following outcomes of communicating science to the public?

|  | Strong negative effect | Negative effect | No effect | Positive effect | Strong positive effect |
| --- | --- | --- | --- | --- | --- |
| Helping the public connect science to everyday lives |  |  |  |  |  |
| Helping the public make informed decisions using science |  |  |  |  |  |
| Informing the public |  |  |  |  |  |
| Exciting the public |  |  |  |  |  |
| Building trust |  |  |  |  |  |

***Demographics***

1. Please indicate your sex:
   1. Female
   2. Male
   3. Other
2. Please indicate your age (in years)
3. What is the highest level of school you have completed or the highest degree you have received?
   1. Less than high school (Grades 1-8 or no formal schooling)
   2. High school graduate (Grade 12 with diploma or GED certificate)
   3. Two year associate degree from a college or university
   4. Four year college or university degree/Bachelor's degree (e.g., BS, BA, AB)
   5. Postgraduate or professional degree, including master's, doctorate, medical or law degree (e.g., MA, MS, PhD, MD, JD, graduate school)
   6. Prefer not to answer
4. Which of the following describes your race. You can select as many as apply.
   1. White
   2. Black or African-American
   3. Asian or Asian-American
   4. Native American/American Indian/Alaska Native
   5. Pacific Islander/Native Hawaiian
   6. Hispanic/Latino
   7. Other
   8. Don't Know
   9. Prefer not to answer
5. Where do you currently reside?
   1. Africa
   2. Antarctica
   3. Asia
   4. Australia
   5. Europe
   6. North America
   7. South America
6. Last year, what was your total family income from all sources, before taxes?
   1. Less than $10,000
   2. $10,000 - $19,999
   3. $20,000 - $29,999
   4. $30,000 - $39,999
   5. $40,000 - $49,999
   6. $50,000 - $74,999
   7. $75,000 - $99,999
   8. $100,000 - $149,999
   9. More than $150,000
   10. Prefer not to answer
